# The mitochondrial genome sequence of *Cyrnea seurati* revealed the phylogenetic relationship in Spiruromorpha and its implications for evolution

**DOI:** 10.1186/s13071-026-07556-1

**Published:** 2026-07-22

**Authors:** Yuan-Ping Deng, Ai-Yun Zhao, Yi-Liu Liu, Yi-Tian Fu, Meng Qi, Guo-Hua Liu

**Affiliations:** 1 College of Animal Science and Technology, Tarim University, Alar, 843300 China; 2 Research Center for Parasites & Vectors, College of Veterinary Medicine, Hunan Agricultural University, Changsha, 410128 Hunan Province China

**Keywords:** Spiruromorpha, *Cyrnea seurati*, Mitochondrial genome, Phylogeny, Vector-borne parasites, Adaptive evolution

## Abstract

**Background:**

The infraorder Spiruromorpha comprises diverse parasitic nematodes of veterinary and medical importance, many of which utilize arthropods as intermediate hosts/vectors. However, phylogenetic relationships and evolutionary history within this group remain poorly resolved due to limitations of single-gene markers and the scarcity of fossil records. Complete mitochondrial genomes offer robust alternatives for resolving deep evolutionary radiations and understanding adaptive processes relevant to parasite–vector–host interactions.

**Results:**

We assembled and characterized the first complete mitochondrial genome of *Cyrnea seurati* (Habronematoidea) from a Eurasian hobby (*Falco subbuteo*) in China. The 13,761 bp circular genome contains 12 protein-coding genes (PCGs), 22 tRNAs, and 2 rRNAs, with gene arrangement conserved within Habronematoidea but featuring unique initiation codons (GTT for *nad4* and TTT for *cytb*). Comparative analysis revealed high genetic divergence from other Habronematoidea species (20.5–37.4% in nucleotide sequences), confirming its distinct generic status. Phylogenomic analyses of 54 Spiruromorpha mitogenomes resolved two major clades and widespread paraphyly among superfamilies, with mitochondrial data providing greater resolution than 18S rRNA. Divergence time estimation, calibrated using published fossil records, traced the most recent common ancestor of Spiruromorpha to the Devonian (~ 366.77 Mya), with major radiations coinciding with the Jurassic–Cretaceous transition—a period marked by diversification of insect vectors and vertebrate hosts. Positive selection was detected in nine PCGs, notably in OXPHOS genes (*cox2*, *cytb*, *nad1*, *nad6*), suggesting adaptive evolution to diverse host environments and metabolic demands. Codon usage analysis revealed strong AT-biased preferences and species-specific adaptive patterns linked to host origins.

**Conclusions:**

This study provides the first mitogenomic resource for the genus *Cyrnea*, significantly advancing the molecular dataset for Spiruromorpha. Our phylogenomic framework resolves long-standing uncertainties in spiruromorph relationships and highlights the potential of mitochondrial markers for tracing parasite–vector coevolutionary dynamics. The detected positive selection signals implicate mitochondrial adaptation in the ecological diversification of these parasites, with implications for understanding nematode parasitism, pathogen control, and the evolutionary interplay between parasites, vectors, and hosts.

**Graphical Abstract:**

**Figure:**
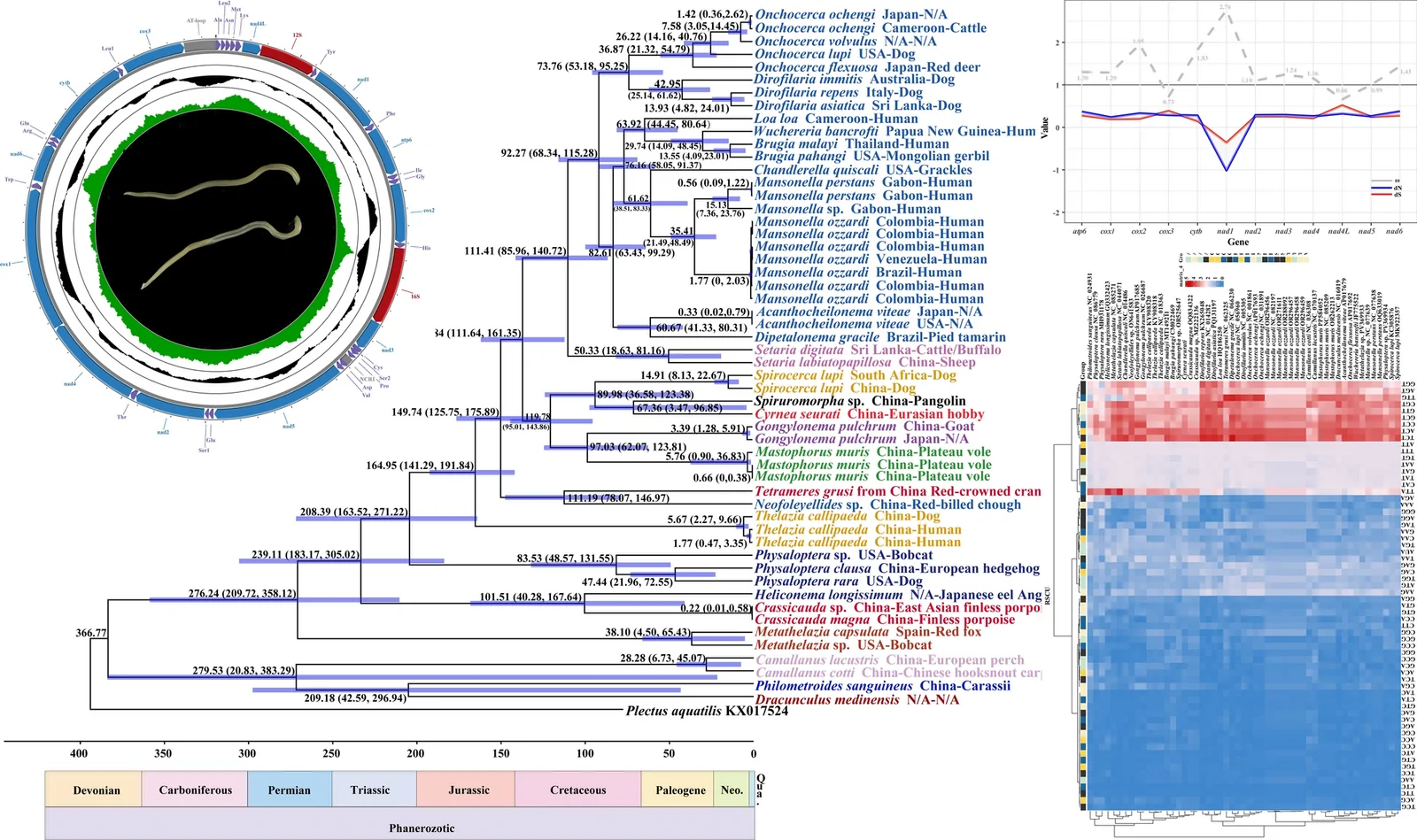

**Supplementary Information:**

The online version contains supplementary material available at https://doi.org/10.1186/s13071-026-07556-1.

## Background

Spiruromorpha parasites represent one of the most successful adaptive radiations within the Nematoda phylum, encompassing numerous species of veterinary and medical significance that utilize arthropods as intermediate hosts [1]. This species-rich and heterogeneous group comprises 10 superfamilies and 312 genera, with an estimated number of species exceeding 3,000 distributed worldwide [2]. Spiruromorpha worms utilize arthropods as intermediate hosts, demonstrating substantial diversification in biological characteristics, and occupy diverse ecological niches—from aquatic to terrestrial environments—and include pathogens affecting domestic animals, wildlife, and humans (e.g., filariasis, trichuriasis) [3–6]. Therefore, understanding their phylogeny is crucial for elucidating the origins of parasitism, host-switching events, and vector-mediated transmission mechanisms, with direct implications for disease control and anthelmintic development [7].

The infraorder Spiruromorpha consists of 10 superfamilies, including Acuarioidea, Aproctoidea, Camallanoidea, Diplotriaenoidea, Filarioidea, Habronematoidea, Physalopteroidea, Rictularioidea, Spiruroidea, and Thelazioidea [8]. While 18S rRNA has been widely used as a molecular marker, it often lacks the resolving power needed for deep phylogenies, yielding conflicting results, including many paraphyletic and polyphyletic families [9–11]. On the other hand, the mitochondrial (mt) *cox1* gene is also frequently used in taxonomic analysis to define phylogenetic relationships in this group, and produces disagreeing results regarding Spiruromorpha relationships [12]. Therefore, analyses based on single-gene markers can produce conflicting results and should be taken with great caution. As a result, currently all published single-gene-based phylogenies lack supporting evidence for the “backbone” of the tree and extremes of evolutionary rate heterogeneity, and many lineages remain poorly resolved. By contrast, phylogenomic analyses based on complete mitochondrial genomes have yielded statistically robust and congruent results that have proven useful in resolving previously controversial phylogenetic relationships across evolutionary lineages [8, 13, 14]. However, representative genomes from key taxa, such as the Habronematoidea genus *Cyrnea*, remain scarce, limiting our understanding of adaptation and diversification in this group.

The utility of fossil records for molecular dating in Spiruromorpha is constrained by the scarcity of unambiguous nematode fossils [15]. The oldest known unambiguous fossil helminths are isolated remains of pentastomids, which are known from the Cambrian–Ordovician [16]. Previous divergence time estimates for spiruromorph nematodes range widely (260.78–28.98 Mya), depending on methodologies and calibration points [17–19]. Moreover, the high diversity and the absence of an informative fossil record have prevented scientists from deriving a consistent evolutionary framework [2, 20–22]. Reconstructing Spiruromorpha’s evolutionary history, therefore, requires integrating multiple constraints: fossil evidence, molecular clock models, and host–vector coevolutionary frameworks.

In this study, we present the first complete mitochondrial genome of *Cyrnea seurati* (Habronematoidea) from China, characterize its genomic features, and conduct comprehensive phylogenomic analyses to resolve Spiruromorpha relationships. Adaptive evolution through analyses of selection pressure and codon usage was further explored with an emphasis on implications for parasite–vector–host interactions and disease transmission.

## Methods

### Sample collection and genomic extraction

Adult specimens of *Cyrnea seurati* were isolated from the ingluvies and intestines of a deceased Eurasian hobby (*Falco subbuteo*) in Beijing, China. Individual male and female nematodes were washed in physiological saline at room temperature. Morphological identification was performed following established keys of *Cyrnea seurati* [23]. Specimens were preserved in 70% ethanol and stored at −20 °C until DNA extraction. Total genomic DNA was extracted using the Wizard^®^ SV Genomic DNA Purification System (Promega, USA) following the manufacturer’s instructions.

### Amplification of partial cox1 and 18S rRNA

Primers used for amplification of *cox1* were designed as previously described: JB3 (5’-TTT TTT GGG CAT CCT GAG GTT TAT-3’) and JB4.5 (5’-TAA AGA AAG AAC ATA ATG AAA ATG-3’), with an approximate length of 440 bp. Primers 18SF (5’-TGT CGG CGG TTA TAT GCT TGT-3’) and 18SR (5’-GCT CGT TAA TGA TCC TTC CGC-3’) were designed by Primer-BLAST in NCBI (https://www.ncbi.nlm.nih.gov/tools/primer-blast/index.cgi) and used to amplify the 18S rRNA sequence of worm samples, with about 1,700 bp in size. Each PCR reaction comprised 25 µL of 2X mix buffer, 2 µL of forward/reverse primer, and 3 µL of DNA template, with nuclease-free water added to bring the total volume to 50 µL. PCR system of the *cox1* gene was performed as Bowles and McManus established [24]. PCR for the 18S gene was carried out in a BIO-RAD T100^™^ thermal cycler (Bio-Rad, Hercules, CA, USA) using the following conditions: pre-denaturation at 95 °C for 1 min, followed by 37 cycles of 98 °C 10 s, 60 °C 30 s, and 72 °C 1 min, with a final extension at 72 °C 8 min. PCR products were subjected to electrophoresis in 1.5% agarose gel, and positive amplicons were purified using NucleoSpin® Gel and PCR Clean-up (Takara Bio USA, Inc.) and sent to BGI Co., Ltd. (Shenzhen, China) for direct sequencing in both directions. The DNA sequences were assembled by DNAMAN v6.0 (Lynnon Biosoft, USA). These sequences were then used in a BLAST search of GenBank to confirm their identities and species identification of the worms of DNA origin.

### Sequence assembly and annotation

High-molecular-weight genomic DNA was sequenced on an Illumina HiSeq 6000 platform (Novogene, China). The complete mitogenome was assembled using Geneious Prime v.2022.0.1 with the partial *cox1* sequence as a reference [25]. The assembly parameters were set to: (i) a minimum overlap identity of 98%; (ii) an overlap ranging from 150 to 200 bp; and (iii) a maximum gap size of 5 bp. Assembly was verified by long-range PCR using five primer pairs (Table S1, Fig. S1). Initial annotation was using the online service MITOs [26]. All 12 protein-coding genes (PCGs, excluding *atp8*) have their initiation/termination codons and gene boundaries determined by the NCBI’s Open Reading Frame (ORF) Finder (https://www.ncbi.nlm.nih.gov/orffinder/). Tandem analysis [27] and sequences of habronematoiid aligning were used to identify the locations and boundaries of two rRNAs (*rrnL* and *rrnS*). Similarly, tRNAscan-SE 2.0 [28] with a threshold score of 1.0 was used to confirm the sequences, orientations, and secondary structures of 22 transfer RNA genes (tRNAs).

### Codon usage bias analysis

For annotated PCGs, the accuracy, amino-acid sequences, and codon usage were inferred and proved by MEGA v.11.0 [29]. Excluding incomplete termination codon T and TA, the relative synonymous codon usage (RSCU) of the total 12 PCGs database was concatenated and calculated using MEGA v.11.0 [29]. The RSCU heatmap of Spiruromorpha visualized usage preferences and represented the RSCU value distribution across synonymous codon families.

### Phylogenetic analysis

Using *Plectus aquatilis* (GenBank No. KX017524) as the outgroup, 53 available mitochondrial sequences of the Spiruromorpha nematodes were acquired from GenBank for phylogenetic analysis (Table 1). Amino-acid sequences of 12 PCGs (excluding the *atp*8) were concatenated and aligned to a single database with MEGA v.11.0, and the data were subjected to Gblocks 0.91b to exclude ambiguity and identify more conserved blocks, with the option for “less stringent” [30]. Using MrBayes 3.2 with the default model [31], Bayesian inference (BI) was conducted with four independent Markov chains by analyzing 10,000,000 generations and sampling trees every 1,000 generations. The most suitable model, “mtMet + F + R5”, was selected based on ModelFinder and applied to perform maximum likelihood (ML) analysis with IQ-Tree v.2.1.3 [32, 33]. In addition, available 18S rRNA sequences within the subfamily Habronematoidea were used to analyze nuclear phylogenetic relationships. The phylogenetic trees were visualized using FigTree v.1.42 (http://tree.bio.ed.ac.uk/software/figtree/).

**Table 1:** Species used to conduct phylogenetic analyses

Superfamily	Family	Species	GenBank No.
Camallanoidea	Camallanidae	*Camallanus lacustris*	NC_070137
		*Camallanus cotti*	NC_036308
Dracunculoidea	Dracunculidae	*Dracunculus medinensis*	NC_016019
	Philometridae	*Philometroides sanguineus*	NC_024931
Filarioidea	Onchocercidae	*Acanthocheilonema viteae*	AP017679
		*Acanthocheilonema viteae*	CM102097
		*Brugia pahangi*	CM022469
		*Brugia malayi*	MT149211
		*Chandlerella quiscali*	NC_014486
		*Dipetalonema gracile*	NC_066230
		*Dirofilaria asiatica*	PQ131197
		*Dirofilaria repens*	KX265048
		*Dirofilaria immitis*	NC_005305
		*Loa loa*	HQ186250
		*Mansonella ozzardi*	NC_082197
		*Mansonella ozzardi*	OR296459
		*Mansonella ozzardi*	OR296458
		*Mansonella ozzardi*	OR296457
		*Mansonella ozzardi*	OR296456
		*Mansonella ozzardi*	OR288092
		*Mansonella ozzardi*	OR271611
		*Mansonella perstans*	NC_077638
		*Mansonella perstans*	OQ633019
		*Mansonella* sp.	NC_077639
		*Neofoleyellides* sp.	ON641583
		*Onchocerca flexuosa*	AP017692
		*Onchocerca lupi*	NC_056960
		*Onchocerca ochengi*	AP017693
		*Onchocerca ochengi*	NC_031891
		*Onchocerca volvulus*	NC_001861
		*Wuchereria bancrofti*	JF775522
	Setaridae	*Setaria digitata*	NC_014282
		*Setaria labiatopapillosa*	NC_044071
Habronematoidea	Tetrameridae	*Crassicauda magnaiii*	OQ834322
		*Crassicauda* sp.	MZ222136
		*Tetrameres grusi*	NC_062325
Physalopteroidea	Physalopteridae	*Heliconema longissimum*	GQ332423
		*Physaloptera clausa*	NC_086779
		*Physaloptera rara*	MH931178
		*Physaloptera* sp.	PV369934
Spiruroidea	Gongylonematidae	*Gongylonema pulchrum*	NC_026687
		*Gongylonema pulchrum*	AP017685
	Spirocercidae	*Mastophorus muris*	NC_085209
		*Mastophorus muris*	PP584052
		*Mastophorus muris*	OR262213
Thelazioidea	Pneumospiruridae	*Metathelazia capsulata*	NC_085271
		*Metathelazia* sp.	PV369933
	*Thelazridae*	*Spirocerca lupi*	MK922357
		*Spirocerca lupi*	KC305876
		*Spiruromorpha* sp.	OR525647
		*Thelazia callipaeda*	NC_018363
		*Thelazia callipaeda*	KY908320
		*Thelazia callipaeda*	KY908318
Outgroup	Plectidae	*Plectus aquatilis*	KX017524

### Divergence time estimation

Sequences from Table 1 were also used to estimate divergence times among Spiruromorpha species. Divergence time estimation was conducted using Bayesian inference implemented in the BEAST v1.10.4 [34]. The most suitable model was determined based on ModelFinder, with “JTT” used as the gamma site model. The molecular clock was calculated based on the fossil dates and with 95% confidence interval (CI). Considering the uncertainty of default calibrations, the fossil calibration following the previous description by Qing et al. [17] synthesizes paleontological and molecular data from published studies [17]. These calibrations were estimated by Qing et al. [17], following a conservative time interval of mean divergence data ranging from 260.79 million years (Mya) to 28.98 Mya in Spiruromorpha: (i) the calibration of the superfamilies Spiruroidea and Filarioidea with an ancient divergence time of 186.51 Mya and 110.67 Mya, respectively; (ii) a calibration for *Camallanus* and *Dracunculus* species of 260.79 Mya was used to represent the potential split time between them; and (iii) *Thelazia callipaeda* species was estimated that the divergence time was 151.90 Mya. Divergence time analysis was set as follows: a random starting tree, a strict clock model, and a birth–death tree prior with an uncorrelated lognormal clock rate [35]. Each analysis was run for 10 million generations with sampling every 1,000 generations, with convergence assessed by the effective sample size (ESS) > 200 in Tracer v1.7.2 [36]. TreeAnnotator v2.6.2 was used to access the posterior probabilities of the maximum clade credibility (MCC) tree with the first 25% tree burn-in [37]. Phylogenetic relationship and divergence time are visualized in FigTree v1.4.3.

### Positive selection detection

Branch-specific *ω* values (dN/dS) for 112 PCGs of the infraorder Spiruromorpha were calculated using the free-ratio model (model = 3, NSsites = 2) in PAML v4.10.10 [38]. This study employed the Branch Model under the maximum likelihood framework to investigate the adaptive evolution mechanisms of mitochondrial genomes in Spiruromorpha worms. Custom R scripts were applied to compute species-specific mean values of ω, dN, and dS (ω = Σω_*i*_ ∕*N*, dN = ΣdN_*i*_ ∕*N*, dS = ΣdS_*i*_ ∕*N*) along evolutionary trajectories from terminal branches to the root node. In addition, MEGA v.11.0 was used to generate genetic distances of 18S rRNA using the alignment database [29].

## Results

### First molecular recognition of *C. seurati* in China

**Fig. 1:**
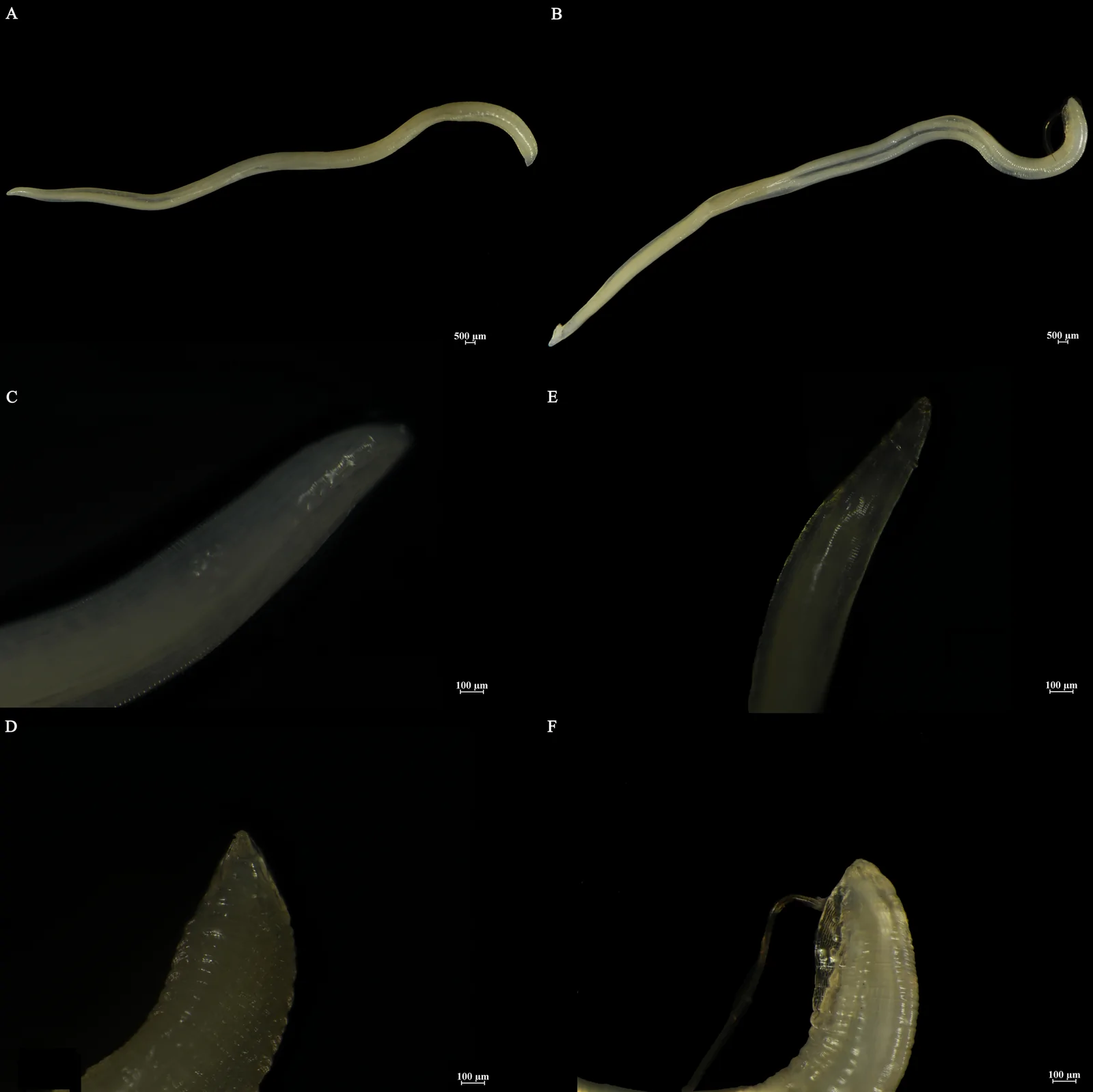
Morphology of *Cyrnea seurati* collected from Eurasian hobby (*Falco subbuteo*) in China. **A** Female species. **B** Male species. **C** Head of the female. **D** Posterior of the female. **E** Head of the male. **F** Caudal purse represented in ventral view

The mt *cox1* sequence of *C. seurati* was amplified and showed 90.1% of similarity with *Mastophorus muris* (GenBank No. MK867480) by BLAST search. Due to a lack of mitochondrial sequences of the genus *Cyrnea*, there were no *cox1* sequences that matched the current species. For the 18S rRNA gene, the sequences of worm samples showed 100% identities with *C. seurati* (GenBank No. EU004816). In general, Europe and North Africa were the primary regions that *C. seurati* inhabits [39, 40]. Based on morphological features (Fig. 1) and molecular evidence (Fig. S2), worms collected from the Eurasian hobby in Beijing were identified as *C. seurati*, which was first recorded in China. Previous data revealed that *C. seurati* can parasitize *F. subbuteo* and red-legged partridge (*Alectoris rufa*) [39, 40]. In this study, the current data also supported its ability to infect *F. subbuteo*.

### Mitogenome characteristics of *C. seurati*

**Fig. 2:**
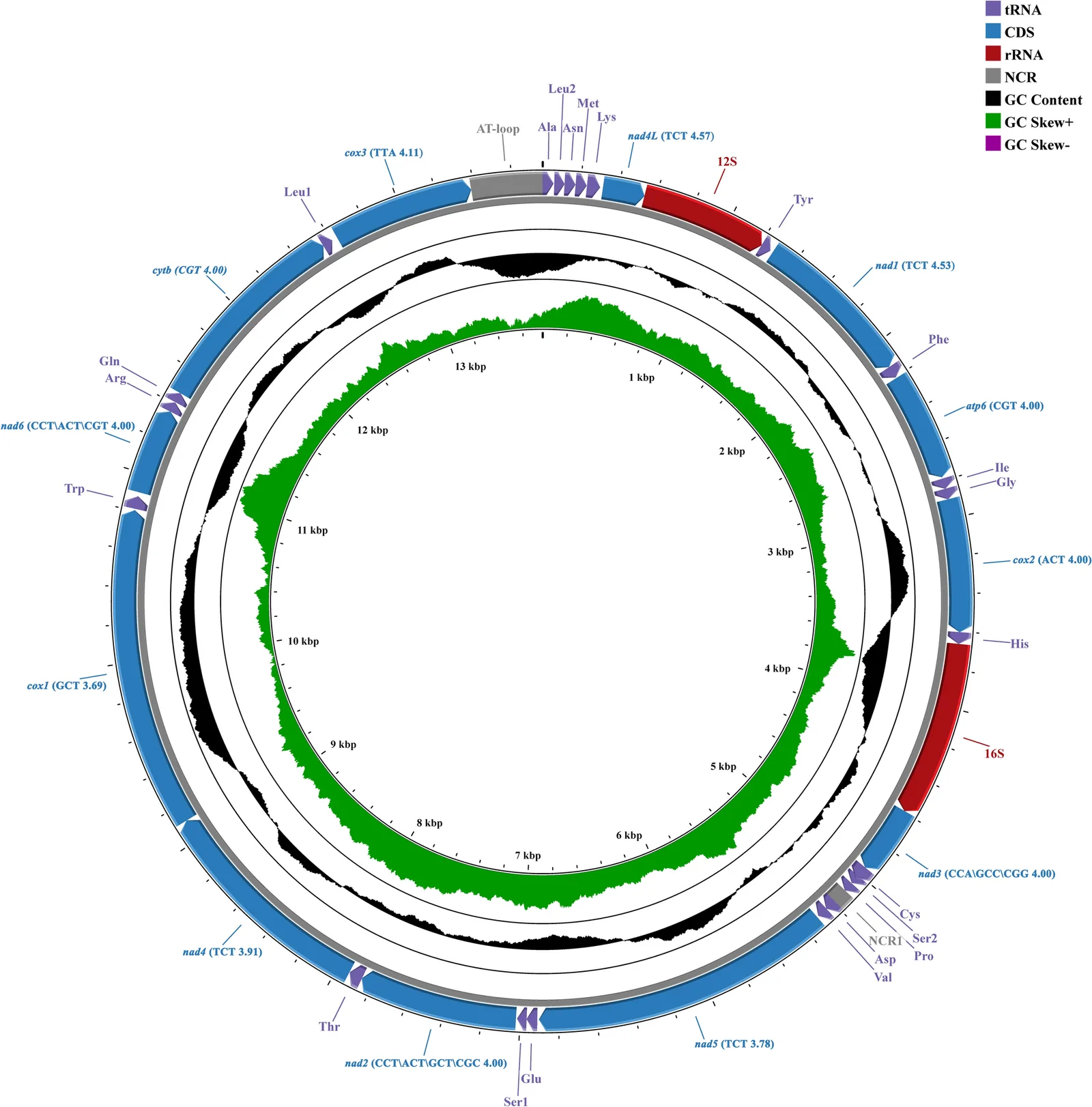
Circular mtDNA genome map with gene metrics. Numbers in parentheses following the 12 protein-coding genes indicate codon usage bias indices

The complete mitogenome was assembled using Geneious Prime with a partial *cox1* sequence as a reference. The genome was a double-stranded structure with a size of 13,761 bp and had been uploaded into the GenBank in the National Center for Biotechnology Information (NCBI) with the accession number PZ102274. The genome comprised 12 PCGs (except the *atp8* gene), 22 transfer RNAs (tRNAs), and 2 rRNAs (*rrnS* and *rrnL*) (Table 2 and Fig. 2), with its arrangement in accordance with Thelazidae (such as *Spirocerca lupi* and *Thelazia callipaeda*), Gongylonematidae (such as *Gongylonema pulchrum*), Onchocercidae (e.g., *Acanthocheilonema viteae* and *Brugia malayi*), Tetrameridae (e.g., *Terameres grusi* and *Crassicauda magna*) [39].

**Table 2:** Organization of the complete mitochondrial genome of this species and estimates of evolutionary divergence between *Cyrnea seurati* and other Habronematoidea species

Gene	Position	Length	No. AA	Ini./Ter	Anticodon	CS/OQ834322		CS/MZ222136		CS/NC_062325	
						NT (%)	AA (%)	NT (%)	AA (%)	NT (%)	AA (%)
tRNA–Ala	1–57	57			TGC	32.6		32.6		34.4	
tRNA–Leu2	62–115	54			TAA	27.0		25.1		27.5	
tRNA–Asn	116–172	57			GTT	27.4		27.4		52.0	
tRNA–Met	174–231	58			CAT	57.8		57.8		22.9	
tRNA–Lys	234–302	69			CTT	42.9		45.6		62.1	
*nad4L*	318–539	222	74	TTG/TAA		30.7	52.1	30.7	52.1	26.7	33.8
*rrnS*	540–1207	668				21.6		21.6		20.7	
tRNA–Tyr	1208–1261	54			GTA	72.0		72.0		51.3	
*nad1*	1277–2137	861	287	TTG/TAG		26.7	37.2	26.7	37.2	24.7	31.1
tRNA–Phe	2139–2202	64			GAA	24.9		31.8		28.9	
*atp6*	2208–2780	573	191	ATA/TAA		27.7	34.2	27.7	34.2	24.4	31.3
tRNA–Ile	2789–2843	55			GAT	36.1		36.2		40.1	
tRNA–Gly	2844–2900	57			TCC	27.3		40.7		28.3	
*cox2*	2901–3602	702	234	TTG/TAA		29.7	42.9	29.7	42.9	29.2	32.6
tRNA–His	3603–3664	62			GTG	34.2		77.5		25.1	
*rrnL*	3665–4591	927				26.9		23.3		23.3	
*nad3*	4592–4952	361	120	ATA/T		37.4	55.4	36.8	56.3	36.5	56.6
tRNA–Cys	4953–5027	75			GCA	41.9		24.9		43.1	
tRNA–Ser2	5010–5059	50			TGA	40.6		40.6		51.7	
tRNA–Pro	5062–5112	51			TGG	31.9		31.9		31.4	
tRNA–Asp	5191–5249	59			GTC	29.8		16.9		22.7	
tRNA–Val	5250–5305	56			TAC	22.9		22.9		47.9	
*nad5*	5315–6901	1587	529	TTG/TAG		26.8	37.3	26.7	37.3	23.0	32.4
tRNA–Glu	6911–6966	56			TTC	32.5		32.5		27.7	
tRNA–Ser1	6967–7016	50			TCT	36.0		36.0		12.0	
*nad2*	7023–7861	839	279	ATT/TA		34.9	53.5	34.9	53.5	27.9	43.0
tRNA–Thr	7862–7932	71			TGT	29.0		27.5		25.0	
*nad4*	7935–9114	1180	393	GTT/T		24.0	33.8	24.0	33.8	23.1	32.8
*cox1*	9115–10803	1689	563	ATA/TAA		24.2	28.3	24.1	28.3	20.5	20.9
tRNA–Trp	10812–10878	67			TCA	21.7		21.7		14.6	
*nad6*	10901–11354	454	151	ATT/T		35.8	61.3	35.8	61.3	27.9	49.3
tRNA–Arg	11355–11408	54			ACG	29.6		29.6		17.2	
tRNA–Gln	11411–11464	54			TTG	25.3		25.3		52.6	
*cytb*	11465–12551	1087	362	TTT/T		24.7	31.4%	24.7	31.4	22.2	28.2
tRNA–Leu1	12552–12605	54			TAG	34.5		34.5		43.1	
*cox3*	12636–13385	750	250	ATA/TAA		34.3	46.6	34.3	46.6	27.9	33.3

OQ834322: *Crassicauda magnaiii*, MZ222136: *Crassicauda* sp., NC_062325: *Tetrameres grusi*

^*^Ini./Ter. initiation/termination codons, *NT* nucleotide, *AA* amino acid, *CS Cyrnea seurati*

Due to the lack of mitochondrial sequence data from the genus *Cyrnea*, boundaries of 12 PCGs were determined by comparing the mitogenome sequence of *C. seurati* with that of species from Habronematoidea. The length of 12 PCGs ranged from 222 to 1,689 bp, with the shortest in the *nad4L* gene and the largest in the *cox1* gene. The other ten PCGs were ranked from longest to shortest as follows: *nad5* (1,587 bp) > *nad4* (1,180 bp) > *cytb* (1,087 bp) > *nad1* (861 bp) > *nad2* (839 bp) > *cox3* (750 bp) > *cox2* (702 bp) > *atp6* (573 bp) > *nad6* (454 bp) > *nad3* (361 bp). Five initiation codons were applied in this mitogenome, including TTG, ATA, GTT, ATT, and TTT. TTG and ATN were popular in ten genes, containing *nad4L*, *nad1*, *cox2*, *nad5*, *atp6*, *nad3*, *cox1*, *nad2*, *nad6*, and *cox3*. Although GTT and TTT were less frequently used as initiation codons, in the current study, they were applied as start codons in *nad4* (GTT) and *cytb* (TTT), respectively. However, in other Habronematoidea species, *Tetrameres grusi* (NC_062325) used TTG as a start in *nad4* and *cytb* genes, and *Crassicauda magna* (OQ834322) and *Crassicauda* sp. (MZ222136) used ATT (*nad4*) and GTG (*cytb*) as their initiations. This difference may be because those species belonged to different genera, suggesting that Habronematoidea species genetic structure sequences are the same, but their genetic compositions are different. Complete termination codons, TAA and TAG, were commonly used in seven PCGs, and incomplete TA and T were also popular in *nad3*, *nad2*, *nad4*, *nad6*, and *cytb* genes (Table 2). The AT content within protein-coding regions was 68.8–81.9%, with AT skews of negative ranging from −0.544 to −0.285. The protein genes of *C. seurati* preferred using G base rather than C base, with GC skews of positive in 0.271–0.732 (Table 3).

**Table 3:** Nucleotide composition and skews of the *Cyrnea seurati* mitochondrial genome

Gene	T (%)	C (%)	A (%)	G (%)	AT (%)	Base skew	
						AT skew	GC skew
*atp6*	55.5	5.8	21.3	17.5	76.8	−0.445	0.504
*cox1*	46.6	11.4	22.2	19.8	68.8	−0.355	0.271
*cox2*	43.7	11.4	24.4	20.5	68.1	−0.285	0.286
*cox3*	49.2	8.8	23.5	18.5	72.7	−0.354	0.356
*cytb*	51.5	8.7	21.9	17.8	73.4	−0.404	0.343
*nad1*	54.4	8.4	18.9	18.4	73.3	−0.483	0.374
*nad2*	57.7	4.6	20.7	16.9	78.4	−0.471	0.569
*nad3*	52.6	5.8	22.2	19.4	74.8	−0.407	0.538
*nad4*	54.7	6.5	20.1	18.7	74.7	−0.463	0.483
*nad4L*	59.5	4.1	17.6	18.9	77.0	−0.544	0.647
*nad5*	56.0	6.4	19.0	18.5	75.0	−0.493	0.485
*nad6*	58.6	2.4	23.3	15.6	81.9	−0.430	0.732
*rrnL*	49.4	6.0	30.9	13.7	80.3	−0.231	0.388
*rrnS*	46.1	7.3	29.0	17.5	75.1	−0.227	0.410
22tRNAs	44.6	5.6	34.2	15.6	78.8	−0.131	0.470
NCR	48.3	5.4	36.1	10.2	84.4	−0.145	0.312

The sizes of all RNAs, including tRNAs and rRNAs, obtained in this study, were 50–78 bp and 668–927 bp, respectively. The predicted secondary structures of fourteen tRNAs identified in this study showed a typical TV loop, in which the TΨC stem loop and variable loop were replaced by a TV-replacement loop structure. This structure was commonly observed in most mitogenomes of nematodes, excluding *Trichinella spiralis* (Owen, 1835) Railliet, 1895 [41–43]. Six tRNAs, including tRNA–His, tRNA–Cys, tRNA–Lys, tRNA–Phe, tRNA–Thr, and tRNA–Trp, were predicted as standard “cloverleaf” structures. The structure of tRNA–Ser^AGN^ and tRNA–Ser^UCN^ lacked DHU-arm/loops, which were replaced by several residues. The AT content in total tRNAs was 78.8%, with an AT skew of −0.131 and GC skew of 0.470, indicating a bias of T and G bases. The *rrnS* (AT skew: −0.227, GC skew: 0.410), located in *nad4L* and tRNA–Tyr, and *rrnL* (AT skew: −0.231, GC skew: 0.388), located between tRNA–His and *nad3*, were also observed this phenomenon.

The highest AT content of 84.4% was in non-coding regions, and AT skew in these regions was negative (−0.145), while the value of GC skew in them was 0.312 (the number of G bases was greatly higher than C bases, Table 3).

### Genetic differences between *C. seurati* and other Habronematoidea species

Comparisons of nucleotide sequences of mitochondrial genes are listed in Table 2, as were amino acids of PCGs. Obvious differences were observed in both 36 genes and the amino-acid sequences of 12 PCGs. General evolutionary divergence of nucleotide and amino acids between *C. seurati* and other Habronematoidea species was 12.0–77.5% and 20.8–61.3% (Table 2), respectively. The least nucleotide difference (12.0%) was present in tRNA–Ser^UCN^ between *C. seurati* and *Tetrameres grusi* (NC_062325), and the largest (77.5%) was observed in tRNA–His between *C. seurati* and *Crassicauda* sp. (MZ222136). The difference of amino-acid sequence was the least in *cox1* (*C. seurati* and *T. grusi*) and the greatest in *nad6* (*C. seurati*, *Crassicauda magna*, and *Crassicauda* sp.). Estimates of nucleotide/amino-acid evolutionary divergence of PCGs between *C. seurati* and *C. magna* (OQ834322), *C. seurati* and *Crassicauda* sp., and *C. seurati* and *T. grusi* were 24.0–37.4%/28.3–61.3%, 24.0–36.8%/28.3–61.3%, and 20.5–36.5%/20.9–56.6% (Table 2), respectively. Three genes (*nad4*, *cox1*, and *cytb*) with nucleotide divergence lower than 25% and only *cox1* with amino-acid divergence lower than 30% were in three genera, *Cyrnea*, *Crassicauda*, and *Tetrameres*, indicating a close evolutionary relationship with these three genes. The differences of rRNAs nucleotide sequences were 23.3–26.9% in *rrnL* and 20.7–21.6% in *rrnS* (Table 2). Comparably, divergences of 22 tRNAs were estimated at 12.0–77.5%. It is acceptable that the divergence of mt DNA sequences in nematode species was 10–20% [44]. The difference observed in the current study in nucleotide sequences of all 12 PCG and two rRNA genes ranged from 20.5 to 37.4%, and the divergence in amino acid was from 20.9 to 61.3%, clearly indicating *Cyrnea seurati* was a distinct species from other Habronematoidea species. The differences among *Cyrnea*, *Crassicauda*, and *Tetrameres* were higher than species-level and further suggested *Cyrnea* was a distinct genus. Compared to higher distances in mitochondrial genes, 18S rRNA may be more conserved in *Cyrnea* and other Habronematoidea species with divergence ranging from 0 to 9.4% (Table 4).

**Table 4:** Species distance based on *p*-distance using 18S rRNA

Species	*C. seurati*	*T. fissipina* EF180077	*C. seurati* EU004816	*C. leptoptera* EU004815	*C. mansioni* AY702701	*M. cloacitectus* EU004814	*C. giliakiana* LC700444	*C. boopis* KY263809	*H. truncata* JX235976	*H. spinigera* HM484351	*S. carolini* DQ503464
*Cyrnea seurati*											
*Tetrameres fissipina* EF180077	0.0709										
*Cyrnea seurati* EU004816	0.0000	0.0711									
*Cyrnea leptoptera* EU004815	0.0129	0.0706	0.0129								
*Cyrnea mansioni* AY702701	0.0120	0.0672	0.0100	0.0041							
*Microtetrameres cloacitectus* EU004814	0.0332	0.0886	0.0332	0.0290	0.0259						
*Crassicauda giliakiana* LC700444	0.0318	0.0809	0.0324	0.0293	0.0270	0.0377					
*Crassicauda boopis* KY263809	0.0335	0.0816	0.0335	0.0303	0.0277	0.0371	0.0012				
*Hadjelia truncata* JX235976	0.0448	0.1018	0.0448	0.0501	0.0436	0.0676	0.0513	0.0500			
*Hedruris spinigera* HM484351	0.0940	0.1434	0.0940	0.1025	0.0991	0.1224	0.1058	0.1076	0.1037		
*Spinitectus carolini* DQ503464	0.0463	0.0986	0.0463	0.0476	0.0450	0.0593	0.0354	0.0354	0.0690	0.1204	

### Adaptive codon usage in Spiruromorpha

**Fig. 3:**
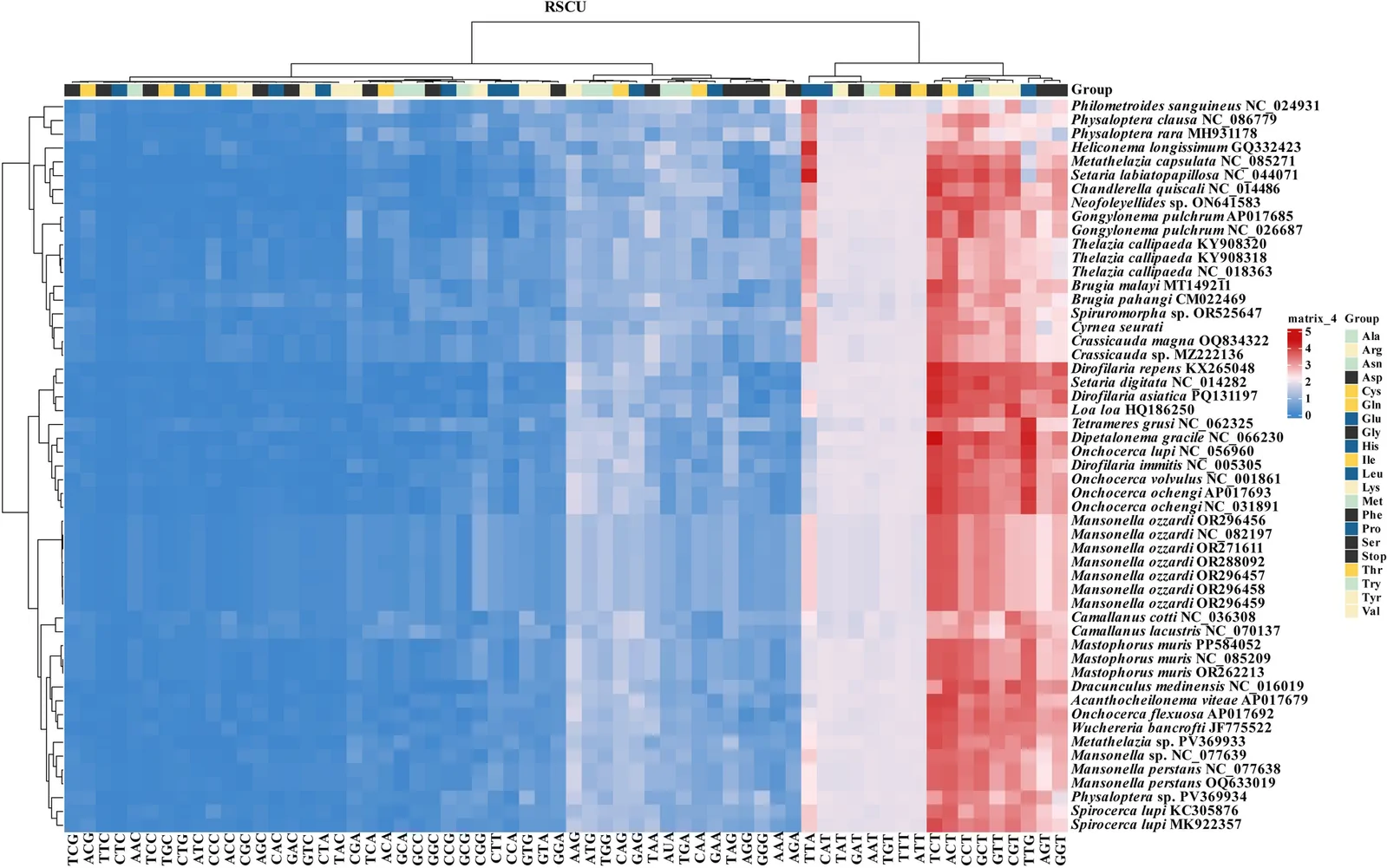
RSCU heatmap showing codon usage patterns variation across Spiruromorpha

Spiruromorpha mitochondrial genomes exhibit both conservation and diversity in synonymous codon usage patterns (RSCU) (Fig. 3). All examined Spiruromorpha species demonstrated absolute preference for the termination codons TAA and TAG, with RSCU in 0.67–1.71 and 0.29–1.33. This represents a conserved molecular synapomorphy in derived Spiruromorpha. Among amino-acid-coding codons, most codons were in the RSCU zone of 0–2, while valine GTT (RSCU = 2.01–3.70), phenylalanine TTT (RSCU = 1.87–2.00), serine TCT (RSCU = 2.61–4.44) and AGT (RSCU = 2.26–3.38, except for *C. seurati* in this study), proline CCT (RSCU = 2.51–3.89, excluding *Philometroides sanguineus* with 1.70), threonine ACT (RSCU = 2.36–4.00), alanine GCT (RSCU = 2.45–4.00), arginine CGT (RSCU = 2.27–4.00), glycine GGT (RSCU = 2.04–3.76) excluding *Thelazia callipaeda* (KY908318) and *Physaloptera* species (PV369934 and MH931178), and leucine TTG (RSCU = 2.22–4.26) except for in *Setaria labiatopapillosa* (NC_044071), *Philometroides sanguineus* (NC_024931), *Heliconema longissimum* (GQ332423) and *Metathelazia capsulata* (NC_085271) lineages exhibited strong positive selection signals, with *Dipetalonema gracile* (NC_066230) showing the peak TCT preference (RSCU = 4.44). The corresponding synonymous codons GTC (RSCU = 0–0.23), CTC (RSCU = 0–0.05), CTA (RSCU = 0–0.36), CTG (RSCU = 0–0.25), TCC (RSCU = 0–0.25), CCC (RSCU = 0–0.47), ACC (RSCU = 0–0.44), ACG (RSCU = 0–0.49), TTC (RSCU = 0–0.13), CGC (RSCU = 0–0.30), and GGC (RSCU = 0–0.56) were also significantly unrepresented. Isoleucine ATT (RSCU = 1.85–2.00), tyrosine TAT (1.67–2.00), histidine CAT (1.52–2.00), asparagine AAT (1.79–2.00), aspartic acid GAT (1.71–2.00), and cysteine TGT (1.73–2.00) also exhibited sustained positive selection patterns with minimal interspecific variation in preference intensity.

Interspecific comparisons reveal adaptive differentiation in critical codon usage. *C. seurati* showed a high preference of methionine ATA codon (RSCU = 1.26), glutamic acid GAA (RSCU = 1.19) and alanine GCT codon (RSCU = 3.14) than other Habronematoidea species (ATA: 0.80 ± 0.32; ATA: 0.81 ± 0.01; GCT: 2.80), while other Habronematoidea species demonstrated stronger usage of proline CCG (RSCU = 0.54 ± 0.08), glutamic acid GAG (RSCU = 1.34 ± 0.14), and serine AGT (RSCU = 2.38 ± 0.08) than that of *C. seurati* (CCG: 0.15; GAG: 0.81; AGT: 1.77). A similar phenomenon was also observed in other species, suggesting that codon selection is linked to species adaptation. Notably, Tetrameridae and Gongylonematidae species displayed termination codon usage with relatively high-frequency TAA (RSCU = 1.71) and a low-frequency TAG (RSCU = 0.29) occurrences. While most synonymous codons exhibit moderate bias, complementary balanced distributions occur in several specific amino acids, namely, methionine ATA (RSCU = 0.41–1.63) versus ATG (RSCU = 0.37–1.59), glutamine CAA (RSCU = 0.35–1.38) versus CAG (RSCU = 0.62–1.65), and tryptophan TGA (RSCU = 0.41–1.48) versus TGG (RSCU = 0.52–1.59). Collectively, RSCU patterns not only reveal core conserved mechanisms of codon optimization in Spiruromorpha mitogenomes, but they also suggest species-specific translational fine-tuning during adaptive evolution.

### Phylogenetic analyses of Spiruromorpha

**Fig. 4:**
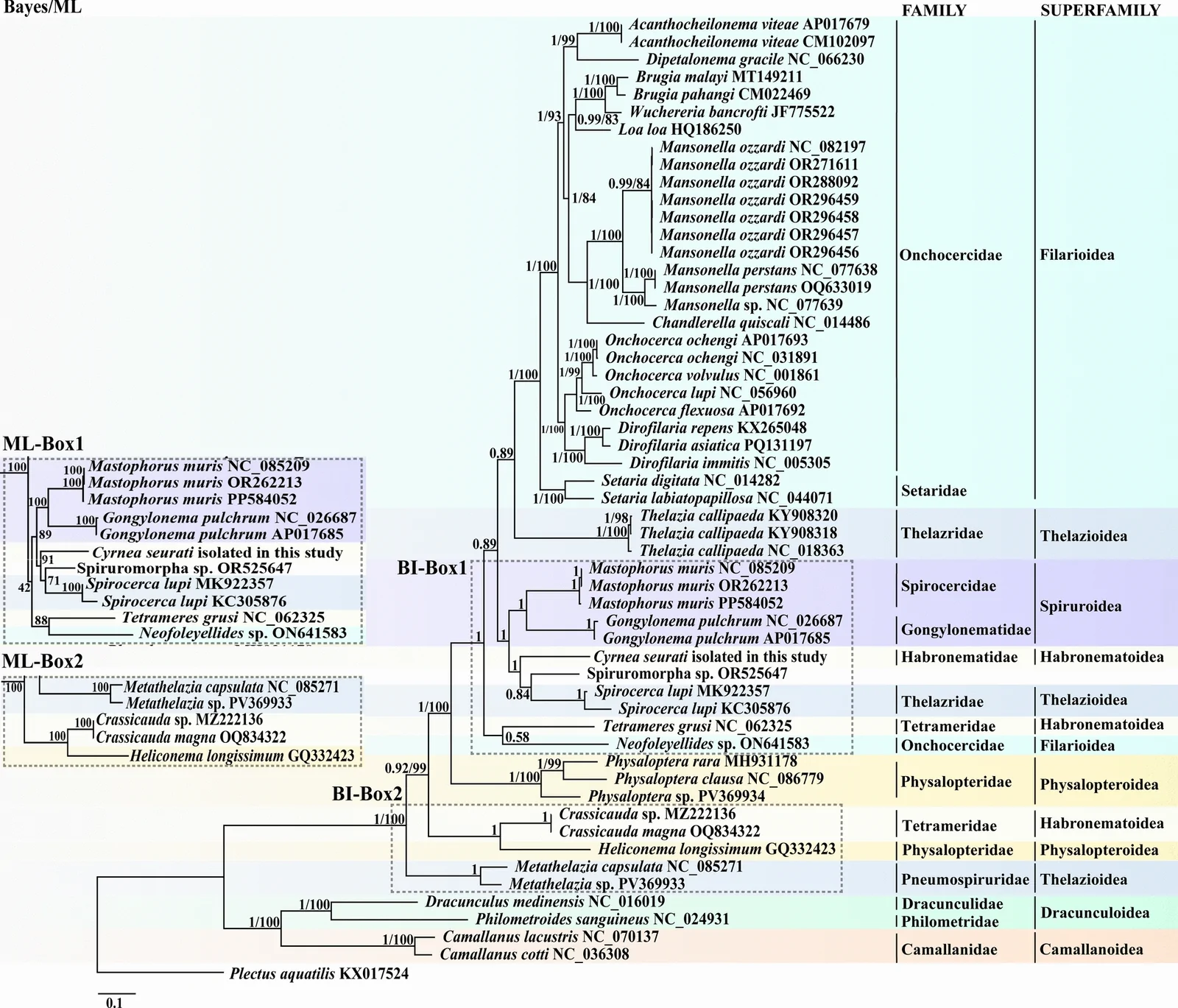
Phylogenetic trees (Bayes and ML) based on 12 PCGs, showing two major clades and widespread paraphyly. Node labels indicate bootstrap values/posterior probabilities. The Box1 and Box2 were two different topologies between Bayes and ML

The 12 PCGs (excluding the *atp8* gene) in the mitogenomes of 54 Spiruromorpha species were used to reconstruct the phylogenetic tree, including 2 species from the superfamily Camallanoidea, 2 species from the superfamily Dracunculoidea, 29 species from the superfamily Filarioidea, 4 species from the superfamily Habronematoidea, 4 species from the superfamily Physalopteroidea, 5 species from the superfamily Spiruroidea, and 8 species from the superfamily Thelazioidea. Phylogenetic trees constructed using BI and ML methods exhibited basically congruent topological frameworks for Spiruromorpha evolutionary relationships, but differed in box 1, box 2, and branch lengths (Fig. 4). Species in ML box 1 grouped a single cluster, but the branch of *T. grusi* and *Neofoleyellides* sp. formed a new cluster in the BI box 1. In box 2, Thelazioidea species were reversed topologically in the BI and ML trees. Comparably, the branch lengths of the BI tree were consistently shorter than the ML tree, indicating that the BI approach may generate more conservative substitution rate estimates at ancestral nodes. Two main clades were grouped in phylogenetic trees: clade I comprising two monophyletic superfamilies, Camallanoidea and Dracunculoidea; and clade II consisting of the remaining four paraphyletic superfamilies (Filarioidea, Habronematoidea, Physalopteroidea, and Thelazioidea) and monophyly Spiruroidea. The paraphyly of families Onchocercidae, Tetrameridae, Physalopteridae, and Thelazidae was fully supported in both trees, with posterior probabilities of higher than 0.58 and ML bootstrap higher than 42 (Fig. 4).

Key discrepancies between BI and ML analyses emerged in the resolution of weakly supported nodes. The clustering of *C. seurati* with Thelaziidae species was more strongly supported in the ML (bootstrap of 91) than BI (posterior probability of 1) analyses, indicating enhanced maximum likelihood sensitivity to evolutionary signals obscured by gene flow in the current study.

Despite overall topological congruence, the ML and BI trees revealed distinct perspectives on phenotype–molecule decoupling. The *C. seurati* and *S. lupi* formed closer taxa in both phylogenetic analyses using 12 PCGs with high BI (posterior probability = 1) and ML support (bootstrap = 91). Their near-identical topological positions reflect robust phylogenetic signals, and the higher probabilistic nature of Spiruromorpha phylogeny by BI estimates may better accommodate ancestral polymorphism. Although the *Cyrnea* and *Tetrameres* species formed closer taxa in both BI and ML using the 18S rRNA gene (Fig. S2), the analyses of mitogenomes revealed relative longer distance from those genera.

### Divergence time estimation in Spiruromorpha

**Fig. 5:**
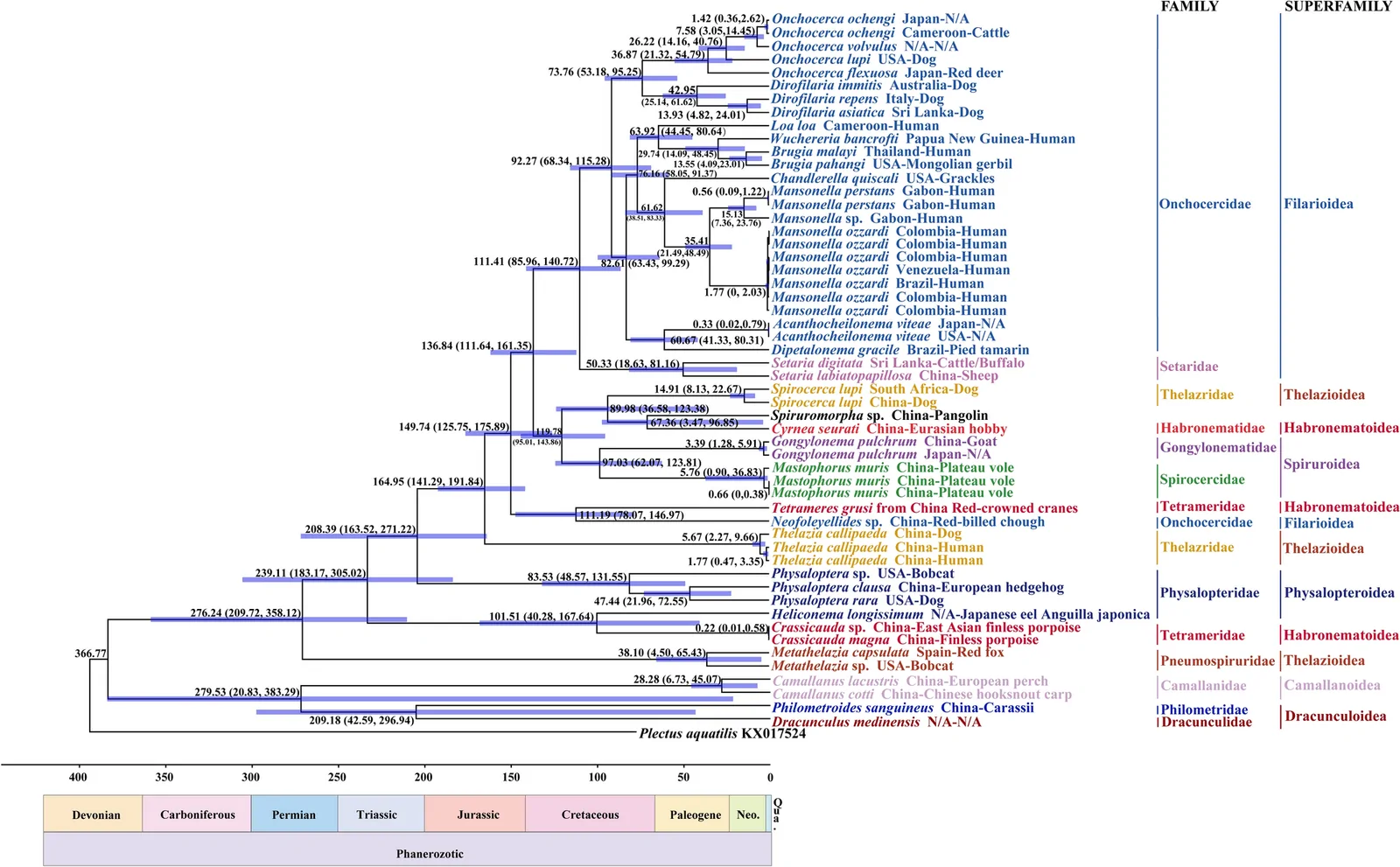
Divergence time estimation for Spiruromorpha calibrated with fossil and molecular (12 mitochondrial PCGs) records. Colored bars indicate different families or superfamilies. Node numbers represent the estimated divergence times integrated from fossil records, and numbers in parentheses represent 95% confidence intervals. Abbreviation in geologic time scale: *Qua.* Quaternary, *Neo.* Neogene

The relative divergence timelines within the infraorder Spiruromorpha were estimated from 0 to 366.77 Mya (Fig. 5), which dates back to a period spanning the end of the Devonian period and the beginning of the Carboniferous. It also suggested that a common ancestor about 366.77 Mya was shared among those seven superfamilies analyzed in this study. Most superfamilies expanded in the late Jurassic and Cretaceous period, and their differentiation times of the five superfamilies were as follows: ~ 279.53 Mya (95% CI 20.83–383.29 Mya) of Camallanoidea, ~ 209.18 Mya (95% CI 42.59–296.94 Mya) of Dracunculoidea, ~ 111.19 Mya (95% CI 78.07–146.97 Mya) of Filarioidea, ~ 119.78 Mya (95% CI 62.07–123.81 Mya) of Spiruroidea, ~ 164.95 Mya (95% CI 141.29–191.84 Mya) of Thelazioidea, ~ 111.19 Mya (95% CI 78.07–146.97 Mya) of Habronematoidea, and ~ 101.51 Mya (95% CI 40.28–167.63 Mya) of Physalopteroidea (Fig. 5).

Monophyletic superfamilies, Camallanoidea and Dracunculoidea, early diverged in the Permian and Triassic periods, respectively; however, there are not enough samples to define the specific differentiation periods of members of these two superfamilies. The stepwise radiation of the superfamily Filarioidea species ranged from 0 to 111.19 Mya, where the divergency time among various genera in the Onchocercidae family was from 29.74 Mya (95% CI 14.09–48.45 Mya) to 92.27 Mya (95% CI 68.34–115.28 Mya), and the time of differentiation among different species within the same genus was 7.58 (95% CI 3.05–14.45 Mya)—42.95 Mya (95% CI 25.15–61.62 Mya). In Spiruromorpha, evolutionary analyses indicated that the paraphyletic Habronematoidea was also one of the early-diverged groups within the intermediate Cretaceous period. For example, the genus *Crassicauda* separated a single group in ~ 101.51 Mya (95% CI 40.28–167.63 Mya), *Tetrameres* species diverged a long distance from ~ 111.19 Mya (95% CI 78.07–146.97 Mya), and *Cyrnea* seurati species underwent niche partitioning ∼ 119.78 Mya (95% CI 95.01–143.86 Mya). The divergence radiation exhibited multiscale factors, including species variation (such as ~ 38.10 Mya (95% CI 4.50–65.43 Mya) divergence between *Metathelazia capsulate* and *Metathelazia* sp.), host origin (such as ~ 5.67 Mya (95% CI 2.27–9.66 Mya) divergence of *T. callipaeda* from dog and human), and environmental responses (for example, ~ 14.91 Mya (95% CI 8.13–22.67 Mya) of *S. lupi* from China and South Africa). The near-zero divergence of the same species collected from the same hosts may reflect that the species originated from a single source and has not had enough time to accumulate genetic differences.

### Selection pressures

**Fig. 6:**
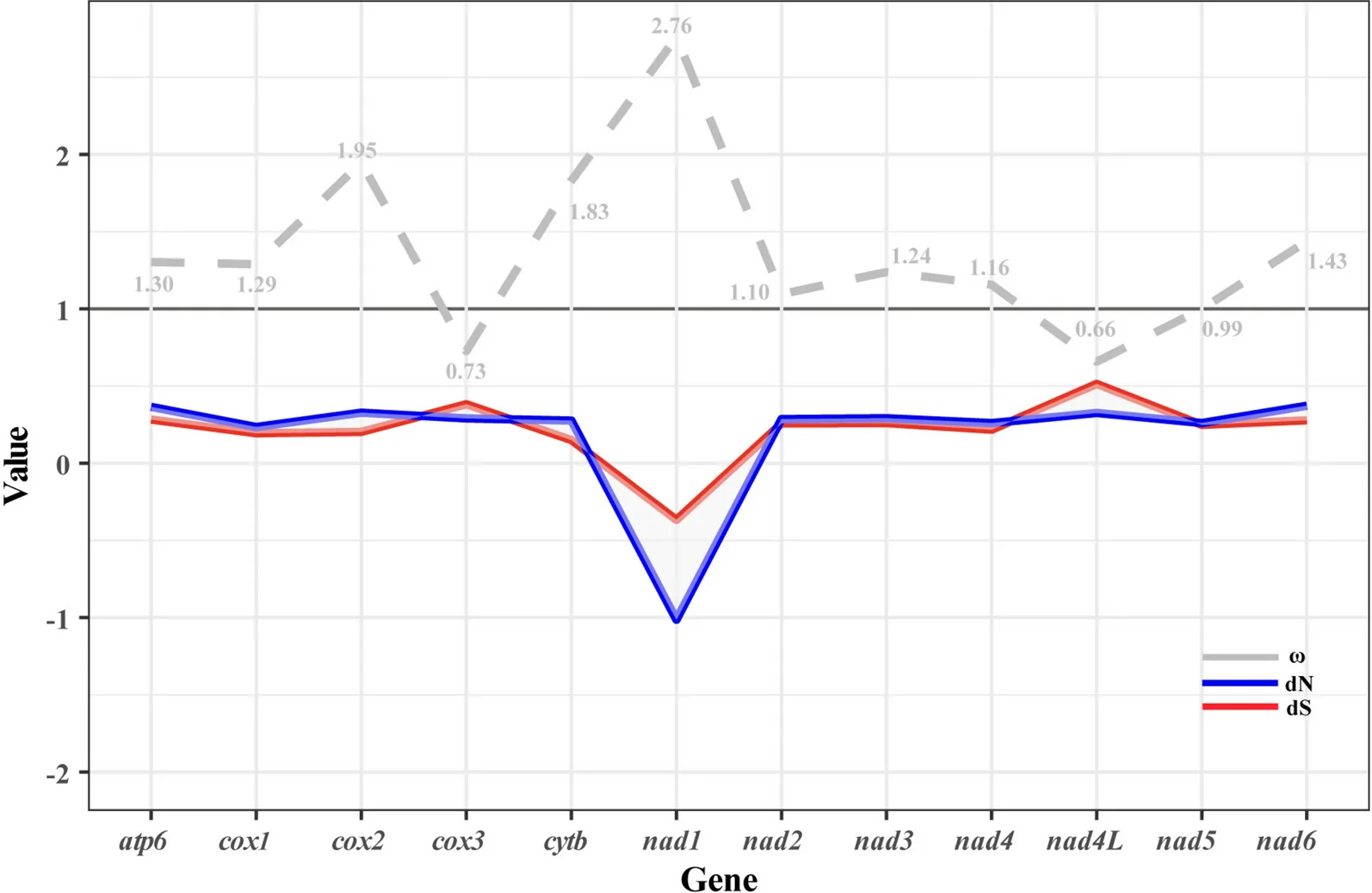
Selection pressures (*ω* values) across 12 PCGs of Spiruromorpha species. Genes with *ω* > 1 are under positive selection; *ω* < 1 under purifying selection

Alignment-free model analyses revealed pervasive negative selection (excluding the *nad1* gene) in spiruromorph mitochondrial genomes (Fig. 6). Likelihood ratio tests with false discovery rate (FDR) correction identified nine genes (*atp6*, *cox1*, *cox2*, *cytb*, *nad1*, *nad2*, *nad3*, *nad4*, and *nad6*) that were under positive selection (*ω* = 1.10–2.76). Genes of *cox2*, *cytb*, *nad1*, and *nad6* (*P* < 0.001) exhibited a strong selection, and *nad1* (*ω* = 2.76) showed the strongest positive selection, indicating their markedly rapid evolutionary rates. There were two genes lower than “1”, *cox3* and *nad4L*, which indicates they are suffering from purifying selection. Only the *ω* rate of the *nad5* gene was “1”, suggesting this gene in Spiruromorpha species suffers from neither positive nor negative selection.

## Discussion

### Mitogenomic features and evolutionary implications

Spiruromorpha mitochondrial genomes are circular and exhibit four types of gene arrangement, mainly manifesting in the rearrangement of the translocation of tRNAs, contrasting with the bipartite structure observed in millipede–parasitic nematode *Ruizia karukerae* of the infraorder Rhigonematomorpha [45]. Comparably, the order and lengths of PCGs in this infraorder are structurally conserved, lacking gene rearrangements [46]. Consistent with most nematodes and the available spiruromorph species [46, 47], the *atp8* gene is absent in *C. seurati* in the current study. Despite structural conservation of PCGs, adaptive codon usage divergences were observed at critical functional sites, wherein *C. seurati* employed GTT and TTT initiation codons for *nad4* and *cytb* genes, instead of the common ATN or TTG [13, 46, 48]. This change mirrors alternative initiation codon strategies in cyrnean mitochondria that may help optimize translation efficiency when parasitizing birds. TAA and incomplete “T” and “TA” were used in termination, indicating the presence of AT-biased selection.

### Codon usage bias and host adaptation

The strong AT-biased codon preferences observed across Spiruromorpha reflect mutational bias and selection for translational efficiency. Similar host-specific codon adaptation has been observed in other parasite groups [49] and may represent a mechanism of host tropism. For example, species of the genera *Crassicauda*, *Mansonella*, *Dirofilaria*, and *Gongylonema* exhibited a similar codon usage model. However, another host-adaptive pattern, in which species collected from different host organisms show different codon usages, was also observed. The differential codon usage between *T. callipaeda* isolates from dogs versus humans further supports this hypothesis and highlights the potential for mitogenomic markers to track host-switching events. Strong positive selection for serine TCT (RSCU = 1.70–4.44, mean 3.60) and threonine ACT (RSCU = 2.36–4.00, mean 3.56) that correlates with AT-biased codon optimization is particularly pronounced in all Spiruromorpha species, including *C. seurati*, with an elevated leucine TTG (mean RSCU 2.98) and alanine GCT (mean RSCU 3.29) preferences, suggesting that these amino acids may be particularly important in mitochondrial proteins under selection. These putative adaptations may enhance species structure and regulate adaptive foraging behavior under worms’ growth and infection, as recorded in *Caenorhabditis elegans* [50, 51].

### Phylogenetic resolution and implications for vector-borne transmission

Phylogenetic analyses of Spiruromorpha revealed basically topological congruence of ML and BI methods for clades (excluding box1 and box2). In both phylogenetic trees, paraphyly is a major feature of most superfamilies in this infraorder, as species from four superfamilies clustered in mixed groups. However, for the superfamilies Camallanoidea and Dracunculoidea, due to the insufficient sample size of complete mitogenomes, it is impossible to determine whether they are polyphyletic, paraphyletic, or monophyletic. Although analyses rejected the monophyletic relationships of superfamilies Thelazioidea and Habronematoidea, consistent with results revealed formerly [1, 48], the topologies of the 18S rRNA tree and mitogenomes were distinct. In this study, the relationship of spiruromorph species was closer to the data based on the whole genomes [17], indicating that mitochondrial datasets amplify algorithm-dependent responses to phylogenetic computation. On the other hand, taxonomies in the current study also presented two major clades: clade I of Camallanoidea and Dracunculoidea, and clade II consisting of Filarioidea, Habronematoidea, Physalopteroidea, Thelazioidea, and Spiruroidea, which is consistent with previous studies [1, 4, 9, 13, 22]. There were a few differences between the current study and other studies using mitogenomes [13, 48] and obvious diversity of species groups based on partial mitogenomes and a single 18S rRNA gene [1, 9, 22], which reveals the structure of phylogenetic trees is more conserved when employing complete mitogenomes, suggesting that mitochondrial data could be more effective and better in systematic analyses and nuclear small subunit ribosomal DNA is an unfit marker in spiruromorph phylogeny [8]. For the difference in mitochondrial performance, the reason may be attributed to the small number of samples used in the latter studies. Despite being members of the Habronematoidea family, the genera *Cyrnea*, *Crassicauda*, and *Tetrameres* do not form a single branch but exhibit a closer relationship with species from other genera with high support. These results indicated a debatable taxonomy of Habronematoidea members. In addition, discrepancies in node support and branch length estimates highlight differences resulting from the methods. For example, in the Filarioidea and Thelazioidea, when the topological structure of the evolutionary tree is consistent, compared with the maximum likelihood method, the Bayesian tree exhibited shorter branch lengths and higher bootstrap nodes, yielding an augmented sensitivity of BI to gene flow signals. Importantly, the close relationship between Habronematoidea and Thelazioidea revealed by mitogenomes may reflect shared evolutionary trajectories linked to similar vector utilization patterns. Many Habronematoidea species utilize orthopterans or coleopterans as intermediate hosts, while Thelazioidea are primarily transmitted via dipterans [3]. Future studies integrating vector mitochondrial barcoding with parasite phylogenetics could test whether host–parasite–vector codiversification shapes these relationships.

### Divergence timing and coevolution with vectors and hosts

Notably, although the number of known nematode fossils is much larger and the majority of nematode lineages are thought to have diverged from the Paleozoic era [15], there are limitations to spiruromorph dating. Comparing the divergence time of spiruromorph species, Spiruromorph divergence timing estimates exhibited possible correlations with several events, such as host changes and habitat transitions. For example, in China, the *T. callipaeda* species from canids and humans diverged about 5.67 Mya, while the species isolated from humans shared a closer common ancestor. The *S. lupi* from South Africa diverged ~ 14.91 Mya from that collected in China. On the other hand, comparing the divergence timeline of Spiruroidea parasites with their hosts [52], the possible origin data matches that of their hosts during the Cretaceous period. The most recent common ancestor of all Filarioidea nematodes is suggested to have existed 111.41–149.74 Mya, coinciding with the transition of the late Jurassic to the middle Cretaceous period [17]. Our analysis indicates that the most recent common ancestor of Dracunculoidea species is ~ 209.18 Mya (95% CI 42.59–296.94 Mya), which is considerably earlier than the ~ 260.79 Mya reported by Qing et al. [17], while both point to a common ancestor in the Triassic period. Compared to co-phylogenetic analyses of Ascaridomorpha based on fossil data from their vertebrate hosts [53], the possible divergence dating result of the member (Spiruromorpha) from Spirurina was also in the Devonian period. This finding is somewhat similar to the split between Spirurina and Plectina in the later Devonian, as inferred from whole-genome amplification (WGA) [17]. These examples not only demonstrate the effectiveness of the mitochondrial genome in determining the evolutionary time of nematodes but also highlight the necessity of using a multidisciplinary approach to verify the divergence age, which involves integrating fossil evidence, conducting concurrent phylogenetic studies, and considering the mutation accumulation rate. However, a great divergence interval existed between Camallanoidea and Dracunculoidea (20.83–383.29 Mya). The possible reasons for this phenomenon were attributed to the small number of available mitogenomes and potential lineage-specific rates in these two superfamilies [54], which emphasizes that more attention should be paid to understanding the evolutionary process in Spiruromorpha species.

The current study estimates that spiruromorph species diverged in the Devonian period, with their most common recent ancestor ~ 366.77 Mya, with major diversification during the Jurassic–Cretaceous transition, a period marked by explosive radiation of insects and vertebrates [52]. This temporal congruence suggests that spiruromorph diversification may have been driven by the availability of new vector and host niches. For example, Filarioidea radiation (~ 111 to 149 Mya) roughly coincides with the diversification of dipteran vectors and mammalian hosts [55–58], while Habronematoidea diversification (~ 101 to 136 Mya) aligns with orthopteran and coleopteran radiations [57, 59, 60]. The relatively recent divergence of Physalopteroidea (~ 101 Mya) may reflect the later colonization of vertebrate hosts. These patterns underscore the need for integrated phylogenomic frameworks that incorporate vector and host evolutionary histories to fully understand parasite diversification.

### Adaptive evolution of mitochondrial genes

The pervasive positive selection signals in the mitochondrial genomes of spiruromorphs are primarily localized to core functional modules of the OXPHOS system, indicating that these electron transport chain components of spiruromorph species may be more tolerant of amino-acid substitutions due to the crucial role of mitochondrial DNA in the electron transport chain and the oxidative phosphorylation (OXPHOS) system [61]. Nematodes may evolve adaptively as a result of this relative easing of selective restrictions, which could aid species in adapting to a variety of ecological niches [60].

Being an essential part of OXPHOS’s electron transport chain, *cytb* is typically quite conserved and has been widely employed in phylogenetics as a neutral evolutionary marker. Nevertheless, data indicate that *cytb* may experience weak positive selection in response to settings with low oxygen or energy-intensive lifestyles, even in the face of substantial functional restrictions [62, 63]. Through a process previously linked to metabolic adaptation, the strong positive selection acting on cytb (a crucial component of Complex III) may improve proton-translocation efficiency via amino-acid substitutions while reducing damage from reactive oxygen species (ROS) [64]. The *nad1* gene is under a lot of selective pressure, which could indicate that it has highly adaptive roles. Selection on these genes may enhance energy production efficiency under hypoxic conditions encountered in host tissues or during vector transmission stages. Otherwise, purifying selection contributes to the acceleration of gene evolution and helps remove harmful mutations in PCGs [65, 66]. In the current investigation, genes *cox3* and *nad4L* exhibit notable selection relaxation. Together, these results demonstrated how important genes involved in mitochondrial energy metabolism are for enabling organismal adaptability to various ecological niches. To separate the niche-specific selection pressures influencing the evolution of the mitogenome in Spirurina, future research combining nuclear-genome data and increased taxonomic sampling is required.

## Conclusions

Synthesizing phylogenetic and selection pressure data, the adaptive radiation of Spiruromorpha emerges as a tripartite process driven by genomic, ecological, and phenotypic interactions. This study presents the first complete mitochondrial genome of *Cyrnea seurati* from China, providing a valuable genomic resource for Spiruromorpha research. Phylogenomic analyses resolve two major clades and reveal widespread paraphyly among superfamilies, challenging current taxonomic frameworks. Divergence time estimates suggest that spiruromorph diversification coincided with Jurassic–Cretaceous radiations of vectors and hosts, implicating coevolutionary processes. Positive selection in OXPHOS genes and host-specific codon usage patterns point to mitochondrial adaptation as a key driver of ecological diversification. These findings advance our understanding of nematode evolution and have implications for disease surveillance and control of vector-borne spiruromorph parasites.

## Supplementary Information

Supplementary material 1: Fig. S1 The verification of the complete mitochondrial genome of the *Cyrnea seurati* species based on long PCR.

Supplementary material 2: Fig. S2 18S rRNA phylogeny of ML and Bayesian of *C. seurati* within Habronematoidea.

Supplementary material 3: Table S1. The primers designed to amplify the mitochondrial genome and verify its correctness

## Data Availability

The data reported in this paper have been deposited in the GenBank in the National Center for Biotechnology Information, under accession number PZ102274, which is publicly accessible at https://www.ncbi.nlm.nih.gov/genbank/.

## References

[1] Choudhury A, Nadler S. Phylogenetic relationships of spiruromorph nematodes (Spirurina: Spiruromorpha) in North American freshwater fishes. J Parasitol. 2018;104:496–504. 10.1645/17-195 29906216

[2] Hodda M. Phylum Nematoda: a classification, catalogue and index of valid genera, with a census of valid species. Zootaxa. 2022;5114:1–289. 10.11646/zootaxa.5114.1.1 35391386

[3] Bezerra-Santos MA, Moroni B, Mendoza-Roldan JA, Perrucci S, Cavicchio P, Cordon R, et al. Wild carnivores and *Thelazia callipaeda* zoonotic eyeworms: a focus on wolves. Int J Parasitol Parasites Wildl. 2022;17:239–43. 10.1016/j.ijppaw.2022.03.005 PMC8924507 35309037

[4] Han L, Yang Y, Lu M, Yu H, Lu Y, Zhou M, et al. Mitogenome phylogenetics of Spiruromorpha porpoise parasite: insights into phylogeny of *Crassicauda magna*. Pathogens. 2024;14:18. 10.3390/pathogens14010018 PMC11768356 39860980

[5] Narapakdeesakul D, Junsiri W, Kongtawee R, Lattisarapunt K, Taweethavonsawat P. Discovery of potentially novel species of the Onchocercidae (Nematoda: Filarioidea) in Burmese fighting chickens (*Gallus gallus*): genetic insights into avian filariasis and co-infection with *Plasmodium juxtanucleare*. Curr Res Parasitol Vector-Borne Dis. 2025;8:100303. 10.1016/j.crpvbd.2025.100303 PMC12395494 40893610

[6] Schroeder JH, McCarthy D, Szestak T, Cook DA, Taylor MJ, Craig AG, et al. *Brugia malayi* microfilariae adhere to human vascular endothelial cells in a C3-dependent manner. PLoS Negl Trop Dis. 2017;11:e0005592. 10.1371/journal.pntd.0005592 PMC5436873 28481947

[7] International Helminth Genomes Consortium. Comparative genomics of the major parasitic worms. Nat Genet. 2019;51:163–74. 10.1038/s41588-018-0262-1 PMC6349046 30397333

[8] Ahmed M, Holovachov O. Twenty years after De Ley and Blaxter—how far did we progress in understanding the phylogeny of the phylum Nematoda? Animals (Basel). 2021;11:3479. 10.3390/ani11123479 PMC8697950 34944255

[9] Černotíková E, Horák A, Moravec F. Phylogenetic relationships of some spirurine nematodes (Nematoda: Chromadorea: Rhabditida: Spirurina) parasitic in fishes inferred from SSU rRNA gene sequences. Folia Parasitol (Praha). 2013;58:135–48. 21776893

[10] Holterman M, Schratzberger M, Helder J. Nematodes as evolutionary commuters between marine, freshwater and terrestrial habitats. Biol J Linn Soc. 2019;128:756–67.

[11] Sokolov SG, Malysheva SV. Molecular characterization of *Ichtyobronema hamulatum* (Moulton, 1931) (Nematoda: Quimperiidae), a common parasite of burbot Lota lota (Linnaeus) (Actinopterygii: Lotidae). Helminthologia. 2017;54:183–8.

[12] Vanstreels RET, Yabsley MJ, Swanepoel L, Stevens KL, Carpenter-Kling T, Ryan PG, et al. Molecular characterization and lesions associated with *Diomedenema diomedeae* (Aproctoidea: Desmidocercidae) from grey-headed albatrosses (*Thalassarche chrysostoma*) on Subantarctic Marion Island. Int J Parasitol Parasites Wildl. 2018;7:155–60. 10.1016/j.ijppaw.2018.04.002 PMC6032498 29988875

[13] Gao JF, Mao RF, Li Y, Sun YY, Gao ZY, Zhang XG, et al. Characterization of the mitochondrial genome of *Tetrameres grusi* and insights into the phylogeny of Spirurina. Int J Parasitol Parasites Wildl. 2022;17:35–42. 10.1016/j.ijppaw.2021.12.001 PMC8688868 34976723

[14] Kim T, Lee Y, Kil HJ, Park J-K. The mitochondrial genome of *Acrobeloides varius* (Cephalobomorpha) confirms non-monophyly of Tylenchina (Nematoda). PeerJ. 2020;8:e9108. 10.7717/peerj.9108 PMC7229770 32440374

[15] Poinar GO. The evolutionary history of nematodes—as revealed in stone, amber and mummies. Leiden: Koninklijke Brill NV; 2011.

[16] Littlewood DTJ, Baets K de. Advances in parasitology. Vol. 90, Fossil parasites. 1st ed. London: Academic Press is an imprint of Elsevier; 2015.

[17] Qing X, Zhang YM, Sun S, Ahmed M, Lo WS, Bert W, et al. Phylogenomic insights into the evolution and origin of Nematoda. Syst Biol. 2025;74:349–58. 10.1093/sysbio/syae073 39737664

[18] Zarlenga DS, Rosenthal BM, La Rosa G, Pozio E, Hoberg EP. Post-Miocene expansion, colonization, and host switching drove speciation among extant nematodes of the archaic genus *Trichinella*. Proc Natl Acad Sci. 2006;103:7354–9. 10.1073/pnas.0602466103 PMC1464345 16651518

[19] Korhonen PK, Pozio E, La Rosa G, Chang BCH, Koehler AV, Hoberg EP, et al. Phylogenomic and biogeographic reconstruction of the *Trichinella* complex. Nat Commun. 2016;7:10513. 10.1038/ncomms10513 PMC4740406 26830005

[20] De Baets K, Dentzien-Dias P, Harrison GWM, Littlewood DTJ, Parry LA. Fossil constraints on the timescale of parasitic helminth evolution. In: De Baets K, Huntley JW, editors. Evol foss rec parasit identif macroevolution parasites. Cham: Springer International Publishing; 2021. p. 231–71.

[21] Hodda M. Phylum Nematoda. Zootaxa. 2007;1668:265–93.

[22] Nadler SA, Carreno RA, Mejía-Madrid H, Ullberg J, Pagan C, Houston R, et al. Molecular phylogeny of clade III nematodes reveals multiple origins of tissue parasitism. Parasitology. 2007;134:1421–42. 10.1017/S0031182007002880 17506928

[23] Lanzhou Veterinary Research Institute, Chinese Academy of Agricultural Sciences. Atlas of parasitic nematodes in humans and animals. 1st ed. Beijing: China Agricultural Science and Technology Press; 2002.

[24] Bowles J, McManus DP. Genetic characterization of the Asian *Taenia*, a newly described taeniid cestode of humans. Am J Trop Med Hyg. 1994;50:33–44. 7905720

[25] Kearse M, Moir R, Wilson A, Stones-Havas S, Cheung M, Sturrock S, et al. Geneious basic: an integrated and extendable desktop software platform for the organization and analysis of sequence data. Bioinformatics. 2012;28:1647–9. 10.1093/bioinformatics/bts199 PMC3371832 22543367

[26] Bernt M, Donath A, Jühling F, Externbrink F, Florentz C, Fritzsch G, et al. MITOS: improved de novo metazoan mitochondrial genome annotation. Mol Phylogenet Evol. 2013;69:313–9. 10.1016/j.ympev.2012.08.023 22982435

[27] Benson G. Tandem repeats finder: a program to analyze DNA sequences. Nucleic Acids Res. 1999;27:573–80. 10.1093/nar/27.2.573 PMC148217 9862982

[28] Chan PP, Lin BY, Mak AJ, Lowe TM. tRNAscan-SE 2.0: improved detection and functional classification of transfer RNA genes. Nucleic Acids Res. 2021;49:9077–96. 10.1093/nar/gkab688 PMC8450103 34417604

[29] Tamura K, Stecher G, Kumar S. MEGA11: molecular evolutionary genetics analysis version 11. Mol Biol Evol. 2021;38:3022–7. 10.1093/molbev/msab120 PMC8233496 33892491

[30] Castresana J. Selection of conserved blocks from multiple alignments for their use in phylogenetic analysis. Mol Biol Evol. 2000;17:540–52. 10.1093/oxfordjournals.molbev.a026334 10742046

[31] Ronquist F, Teslenko M, van der Mark P, Ayres DL, Darling A, Höhna S, et al. MrBayes 3.2: efficient Bayesian phylogenetic inference and model choice across a large model space. Syst Biol. 2012;61:539–42. 10.1093/sysbio/sys029 PMC3329765 22357727

[32] Kalyaanamoorthy S, Minh BQ, Wong TKF, von Haeseler A, Jermiin LS. Modelfinder: fast model selection for accurate phylogenetic estimates. Nat Methods. 2017;14:587–9. 10.1038/nmeth.4285 PMC5453245 28481363

[33] Minh BQ, Schmidt HA, Chernomor O, Schrempf D, Woodhams MD, von Haeseler A, et al. IQ-TREE 2: new models and efficient methods for phylogenetic inference in the genomic era. Mol Biol Evol. 2020;37:1530–4. 10.1093/molbev/msaa015 PMC7182206 32011700

[34] Suchard MA, Lemey P, Baele G, Ayres DL, Drummond AJ, Rambaut A. Bayesian phylogenetic and phylodynamic data integration using BEAST 1.10. Virus Evol. 2018;4:vey016. 10.1093/ve/vey016 PMC6007674 29942656

[35] Drummond AJ, Ho SYW, Phillips MJ, Rambaut A. Relaxed phylogenetics and dating with confidence. PLoS Biol. 2006;4:e88. 10.1371/journal.pbio.0040088 PMC1395354 16683862

[36] Rambaut A, Drummond AJ, Xie D, Baele G, Suchard MA. Posterior summarization in Bayesian phylogenetics using tracer 1.7. Syst Biol. 2018;67:901–4. 10.1093/sysbio/syy032 PMC6101584 29718447

[37] Dellicour S, Gill MS, Faria NR, Rambaut A, Pybus OG, Suchard MA, et al. Relax, keep walking - a practical guide to continuous phylogeographic inference with BEAST. Mol Biol Evol. 2021;38:3486–93. 10.1093/molbev/msab031 PMC8321535 33528560

[38] Yang Z. PAML 4: phylogenetic analysis by maximum likelihood. Mol Biol Evol. 2007;24:1586–91. 10.1093/molbev/msm088 17483113

[39] Honisch M, Krone O. Phylogenetic relationships of Spiruromorpha from birds of prey based on 18S rDNA. J Helminthol. 2008;82:129–33. 10.1017/S0022149X08912359 18252018

[40] Millán J, Gortázar C, Villafuerte R. Ecology of nematode parasitism in red-legged partridges (*Alectoris rufa*) in Spain. Helminthologia. 2004;41:33–7.

[41] Clary DO, Wolstenholme DR. The mitochondrial DNA molecular of *Drosophila yakuba*: nucleotide sequence, gene organization, and genetic code. J Mol Evol. 1985;22:252–71. 10.1007/BF02099755 3001325

[42] He Y, Jones J, Armstrong M, Lamberti F, Moens M. The mitochondrial genome of *Xiphinema americanum *sensu stricto (Nematoda: Enoplea): considerable economization in the length and structural features of encoded genes. J Mol Evol. 2005;61:819–33. 10.1007/s00239-005-0102-7 16315110

[43] Lavrov DV, Brown WM. *Trichinella spiralis* mtDNA: a nematode mitochondrial genome that encodes a putative ATP8 and normally structured tRNAs and has a gene arrangement relatable to those of coelomate metazoans. Genetics. 2001;157:621–37. 10.1093/genetics/157.2.621 PMC1461501 11156984

[44] Blouin MS. Molecular prospecting for cryptic species of nematodes: mitochondrial DNA versus internal transcribed spacer. Int J Parasitol. 2002;32:527–31. 10.1016/s0020-7519(01)00357-5 11943225

[45] Kim T, Kern E, Park C, Nadler SA, Bae YJ, Park JK. The bipartite mitochondrial genome of *Ruizia karukerae* (Rhigonematomorpha, Nematoda). Sci Rep. 2018;8:7482. 10.1038/s41598-018-25759-0 PMC5945635 29749383

[46] Li H, Chen W, Qi W, Ren Z, Pan X, Shen F, et al. Molecular characterization of a novel Spiruromorpha species in wild Chinese pangolin by mitogenome sequence analysis. Parasitol Res. 2024;123:137. 10.1007/s00436-024-08143-y 38376760

[47] Deng YP, Li R, Wang HM, Liu GH, Tu Y. Complete mitochondrial genome of *Contracaecum* sp. (Nematoda: Ascarididae) from night herons in China. J Nematol. 2022;54:20220048. 10.2478/jofnem-2022-0048 PMC9676673 36457371

[48] Aleix-Mata G, Arcenillas-Hernández I, de Ybáñez MRR, Martínez-Carrasco C, Montiel EE, Sánchez A. Complete mitochondrial genome of *Metathelazia capsulata* (Pneumospiruridae) and comparison with other Spiruromorpha species. Parasitol Res. 2023;123:3. 10.1007/s00436-023-08035-7 38047982

[49] Mitreva M, Wendl MC, Martin J, Wylie T, Yin Y, Larson A, et al. Codon usage patterns in Nematoda: analysis based on over 25 million codons in thirty-two species. Genome Biol. 2006;7:R75. 10.1186/gb-2006-7-8-r75 PMC1779591 26271136

[50] Edwards C, Canfield J, Copes N, Brito A, Rehan M, Lipps D, et al. Mechanisms of amino acid-mediated lifespan extension in *Caenorhabditis elegans*. BMC Genet. 2015;16:8. 10.1186/s12863-015-0167-2 PMC4328591 25643626

[51] Saitoh Y, Katane M, Miyamoto T, Sekine M, Sakai-Kato K, Homma H. d-Serine and d-alanine regulate adaptive foraging behavior in *Caenorhabditis elegans* via the NMDA receptor. J Neurosci. 2020;40:7531–44. 10.1523/JNEUROSCI.2358-19.2020 PMC7511192 32855271

[52] Kumar S, Stecher G, Suleski M, Hedges SB. TimeTree: a resource for timelines, timetrees, and divergence times. Mol Biol Evol. 2017;34:1812–9. 10.1093/molbev/msx116 28387841

[53] Li L, Lü L, Nadler SA, Gibson DI, Zhang LP, Chen HX, et al. Molecular phylogeny and dating reveal a terrestrial origin in the early carboniferous for ascaridoid nematodes. Syst Biol. 2018;67:888–900. 10.1093/sysbio/syy018 29528459

[54] Carruthers T, Sanderson MJ, Scotland RW. The implications of lineage-specific rates for divergence time estimation. Syst Biol. 2020;69:660–70. 10.1093/sysbio/syz080 PMC7302051 31808929

[55] Sarwar M. Typical flies: natural history, lifestyle and diversity of Diptera. In Life cycle and development of Diptera. London: IntechOpen; 2020.

[56] Zhang Q, Wang B. Evolution of lower brachyceran flies (Diptera) and their adaptive radiation with angiosperms. Front Plant Sci. 2017. 10.3389/fpls.2017.00631. PMC5401883 28484485

[57] Peris D, Condamine FL. The angiosperm radiation played a dual role in the diversification of insects and insect pollinators. Nat Commun. 2024;15:552. 10.1038/s41467-024-44784-4 PMC10803743 38253644

[58] Soghigian J, Sither C, Justi SA, Morinaga G, Cassel BK, Vitek CJ, et al. Phylogenomics reveals the history of host use in mosquitoes. Nat Commun. 2023;14:6252. 10.1038/s41467-023-41764-y PMC10558525 37803007

[59] Beutel RG, Xu C, Jarzembowski E, Kundrata R, Boudinot BE, McKenna DD, et al. The evolutionary history of Coleoptera (Insecta) in the late Palaeozoic and the Mesozoic. Syst Entomol. 2024;49:355–88.

[60] Chang H, Qiu Z, Yuan H, Wang X, Li X, Sun H, et al. Evolutionary rates of and selective constraints on the mitochondrial genomes of Orthoptera insects with different wing types. Mol Phylogenet Evol. 2020;145:106734. 10.1016/j.ympev.2020.106734 31972240

[61] Pandey A, Suman S, Chandna S. Predictive role of mitochondrial genome in the stress resistance of insects and nematodes. Bioinformation. 2010;5:21–7. 10.6026/97320630005021 PMC3040000 21346874

[62] Bondareva OV, Potapova NA, Konovalov KA, Petrova TV, Abramson NI. Searching for signatures of positive selection in cytochrome b gene associated with subterranean lifestyle in fast-evolving arvicolines (Arvicolinae, Cricetidae, Rodentia). BMC Ecol Evol. 2021;21:92. 10.1186/s12862-021-01819-4 PMC8136191 34016058

[63] da Fonseca RR, Johnson WE, O’Brien SJ, Ramos MJ, Antunes A. The adaptive evolution of the mammalian mitochondrial genome. BMC Genomics. 2008;9:119. 10.1186/1471-2164-9-119 PMC2375446 18318906

[64] Vercellino I, Sazanov LA. The assembly, regulation and function of the mitochondrial respiratory chain. Nat Rev Mol Cell Biol. 2022;23:141–61. 10.1038/s41580-021-00415-0 34621061

[65] Ometto L, Li M, Bresadola L, Varotto C. Rates of evolution in stress-related genes are associated with habitat preference in two *Cardamine* lineages. BMC Evol Biol. 2012;12:7. 10.1186/1471-2148-12-7 PMC3398273 22257588

[66] Stewart JB, Freyer C, Elson JL, Wredenberg A, Cansu Z, Trifunovic A, et al. Strong purifying selection in transmission of mammalian mitochondrial DNA. PLoS Biol. 2008;6:e10. 10.1371/journal.pbio.0060010 PMC2214808 18232733

