# Supplementary material for: The mitochondrial genome sequence of Cyrnea seurati revealed the phylogenetic relationship in Spiruromorpha and its implications for evolution
Source: Parasit Vectors. 2026 Jul 22;19:451. doi: 10.1186/s13071-026-07556-1 (PMC13679273; doi:10.1186/s13071-026-07556-1)
Supplement: Supplementary file 1 [file 13071_2026_7556_MOESM3_ESM.docx]

**Table S1**. The primers designed to amplify the mitochondrial genome and verify its correctness.

| Primer Name | Sequence (5’ – 3’) | Position | Length |
| --- | --- | --- | --- |
| YSF1 | AGCCCTCCTTATTAGATTAGTT | 900-3492 bp | 2593 bp |
| YSR1 | CATAATACAATCCGACTAAAGG |  |  |
| YSF2 | TTTAGTCGGATTGTATTATGGTCAG | 3473-6468 bp | 2996 bp |
| YSR2 | CCTACACGAAACAAATAAATCAAGC |  |  |
| YSF3 | GGGGTTCTTGTAGGAAGGAGTA | 6330-9649 bp | 3320 bp |
| YSR3 | TTCCCACTGTATGAAGACCTAA |  |  |
| YSF4 | GGGAATCCTTTATTGTATCAGC | 9862-12218 bp | 2357 bp |
| YSR4 | TAGCCAAAGAATCAGACTCCACAA |  |  |
| YSF5 | AGTTATTGGGCGGCTGTGGTTA | 11846-660 bp | 2576 bp |
| YSR5 | AGCCGATTATTCTGGAACACTATTT |  |  |
